# Development of an explainable machine learning model for predicting the occurrence of advanced diabetic kidney disease

**DOI:** 10.3389/fendo.2026.1722013

**Published:** 2026-04-02

**Authors:** Kaiwen Zheng, Lun Liu, Jianbin You, Jingyi Su, Ling Li, Jiahao Chen, Liangyuan Chen, Yanfen Zeng, Jinhua Chen, Gang Chen, Junping Wen

**Affiliations:** 1 Department of Clinical Laboratory, Fuzhou University Affiliated Provincial Hospital, Fuzhou, China; 2 Shengli Clinical Medical College of Fujian Medical University, Fujian Provincial Hospital, Fujian Medical University, Fuzhou, China; 3 Department of Laboratory Medicine, The First Affiliated Hospital of Xiamen University, Xiamen Key Laboratory of Genetic Testing, Xiamen, China; 4 Department of Endocrinology, Fuzhou University Affiliated Provincial Hospital, Fuzhou, China

**Keywords:** advanced diabetic kidney disease, explainable, machine learning, partial dependence plot, prediction model, SHapley Additive exPlanation

## Abstract

**Aims:**

This study aims to develop an interpretable machine learning (ML) model for predicting the occurrence of advanced diabetic kidney disease (DKD), with the objective of identifying patients at an early stage of the disease, thereby facilitating timely and appropriate clinical intervention.

**Methods:**

Variable selection was performed using a combination of the least absolute shrinkage and selection operator (LASSO) and recursive feature elimination (RFE) techniques. A prediction model was constructed and validated using eight ML algorithms, and the model’s performance was evaluated using area under curve (AUC), accuracy, sensitivity, specificity, positive predictive value (PPV), negative predictive value (NPV), F1 score, Brier score, calibration curve, and decision curve analysis (DCA). The SHapley Additive exPlanation (SHAP) and partial dependence plot (PDP) methods were employed to interpret the model both locally and globally. Finally, the prediction model was integrated into a network platform based on the Shiny application for direct use by clinicians and patients.

**Results:**

Serum creatinine, age, hemoglobin, serum urea, serum ALP, serum UA, platelet count, serum osmolality, serum bicarbonate, and monocyte count were identified as the most important variables in the advanced DKD model. Eight ML models were developed using these five variables. Among them, the logistic regression (LR) model demonstrated accurate predictive ability in both internal and external validation, with AUCs of 0.948 (95%CI: 0.920-0.975) and 0.898 (95%CI: 0.883-0.913), respectively. Furthermore, the LR model exhibited excellent performance in terms of accuracy, sensitivity, PPV, NPV, F1 score, and Brier score. The results of the calibration curve and DCA also indicate a high degree of consistency between the predicted and observed risks of the RF model, with a net return approaching full coverage. The model developed is available through LR-based online calculators for clinicians, free of charge: https://dev2333.shinyapps.io/logistics1/.

**Conclusion:**

This study developed and validated an interpretable LR model for predicting the occurrence of advanced DKD. The LR model can assist clinical practice by effectively identifying individuals at higher risk of advanced DKD at an early stage, allowing patients to receive timely and personalized treatment, and thereby providing a reliable foundation for improving patient prognosis and optimizing medical resource utilization.

## Introduction

Diabetic kidney disease (DKD), as one of the most severe microvascular complications of diabetes, has emerged as the leading cause of end-stage renal disease (ESRD) worldwide (1). According to the most recent report from the International Diabetes Federation, over 10.5% (536.6 million individuals) of adults globally are affected by diabetes, a figure projected to rise to 12.2% (783.2 million individuals) by 2045 (2). As the prevalence of diabetes continues to increase, the number of DKD cases worldwide has seen an explosive surge. Approximately 30-40% of diabetic patients will develop DKD, with about 50% progressing to ESRD, ultimately facing renal failure and requiring renal replacement therapy (RRT) (3). It is projected that by 2030, global RRT usage will more than double, increasing from 2.618 million individuals in 2010 to 5.439 million (4). Although RRT saves lives, its expanded use undeniably imposes a substantial economic burden on countries globally, particularly in many low- and middle-income nations. Consequently, early identification of high-risk patients in the middle and late stages of DKD, coupled with targeted interventions, has become essential for improving DKD prognosis and enhancing cost-effectiveness across nations.

Although several commonly used indicators, such as estimated glomerular filtration rate (eGFR) and urine albumin/creatinine ratio (UACR), are employed to assess the risk of DKD, they exhibit limitations in the early detection of advanced DKD, including low sensitivity and specificity, as well as a narrow detection window (5–7). For example, eGFR may underestimate the extent of kidney damage during early screening and fail to promptly detect minor changes in kidney function (8). Some patients with DKD may not exhibit proteinuria during the early stages of the disease or even as the disease progresses, resulting in false-negative results and, consequently, a missed diagnosis (9). Therefore, there is a pressing need to develop more sensitive and precise predictive tools to enable timely intervention while diabetic nephropathy remains in the subclinical stage.

With the rapid advancement of machine learning (ML) and artificial intelligence, particularly their widespread application in the medical field, numerous studies have begun to investigate the prediction of the onset and progression of DKD using ML. Chan et al. developed the KidneyIntelX model to predict advanced DKD using the random forest (RF) algorithm (10). Although it achieved a relatively high negative predictive value (NPV) of 0.9, its area under curve (AUC) was only 0.77, which remains unsatisfactory. Another approach involves constructing a recurrent neural network model by utilizing electronic medical records (EMRs) (11). This model demonstrates stable and high prediction accuracy for advanced DKD, but its interpretability is limited. The ML model developed by Zou et al. successfully predicted the risk of ESRD in patients with DKD (12). However, this study did not account for the validation of multi-center data. This suggests that while the development of ML models for predicting the risk of advanced DKD has yielded preliminary results, challenges remain, particularly regarding the interpretability of the models and the reliability of multi-center validation.

The present study aims to develop and validate an interpretable ML model for forecasting the occurrence of advanced DKD, utilizing a multi-center dataset, ML algorithms, SHapley Additive exPlanation (SHAP) and partial dependence plot (PDP) methods, to facilitate the early identification of patients with advanced DKD, thereby enabling timely and appropriate clinical intervention.

## Methods

### Study design and population

**Figure 1:**
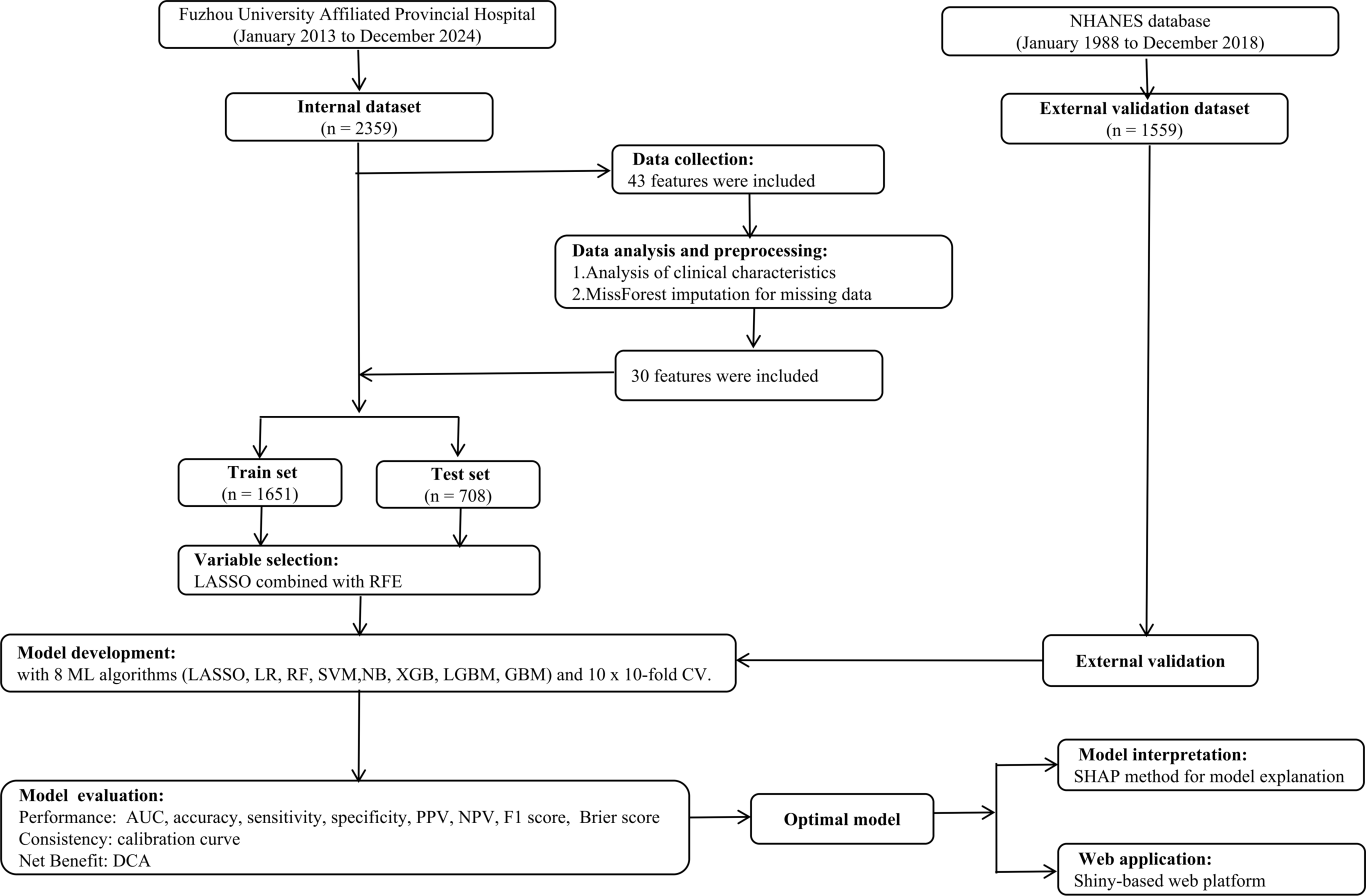
Flow chart of the study design. NHANES, national health and nutrition examination survey; LASSO, least absolute shrinkage and selection operator; XGB, eXtreme gradient boosting; RF, random forest; RFE, recursive feature elimination; LR, logistic regression; SVM, support vector machine; NB, Naive Bayes; LGBM, light gradient boosting machine; GBM, gradient boosting machine; CV, cross-validation; AUC, area under the receiver operating characteristic curve; PPV, positive predictive value; NPV, negative predictive value; DCA, decision curve analysis; SHAP, SHapley Additive exPlanation.

Details of the study design are shown in **Figure 1**. The present study collected data from 2359 patients diagnosed with DKD, admitted to Fuzhou University Affiliated Provincial Hospital between January 2013 and December 2024, forming the internal dataset. The inclusion criteria were as follows: (1) fasting plasma glucose (FPG) ≥ 126 mg/dL (7.0 mmol/L), or 2-h plasma glucose (2-h PG) ≥ 200 mg/dL (11.1 mmol/L) during the oral glucose tolerance test (OGTT), or hemoglobin A1c (HbA1c) ≥ 6.5% (48 mmol/mol), or random plasma glucose ≥ 200 mg/dL (11.1 mmol/L) (13); (2) for a minimum of 3 months, the presence of either of the following: albumin-to-creatinine ratio (ACR) ≥ 30 mg/g (3 mg/mmol), urine sediment abnormalities, persistent hematuria, electrolyte and other abnormalities caused by tubular disorders, histological abnormalities, structural abnormalities identified through imaging, or a history of kidney transplantation (14); (3) patients aged ≥ 18 years. The definitions of the various stages of chronic kidney disease (CKD) were based on the eGFR categories (G1–G5) (14). Based on the CKD stage, all patients were categorized into early DKD (G1-G2, n = 223) and advanced DKD (G3-G5, n = 2136). The study protocol adhered to the guidelines of the Declaration of Helsinki and was approved by the Ethics Committee of Fuzhou University Affiliated Provincial Hospital (K2025-02-116). This study was retrospective, and all data were anonymized, thus waiving the requirement for informed consent from patients.

### Data collection

In this study, data from 43 variables were extracted from the EMR of inpatients, which included the following: (1) demographic information: age, gender, alcohol consumption history, smoking history, and BMI; (2) comorbidities: hypertension, anemia, heart failure, malignancy; (3) laboratory indicators: serum urea, serum uric acid (UA), serum inorganic phosphate (IP), serum creatinine, serum calcium, serum albumin, serum bicarbonate, serum glucose, neutrophil count, white blood cell (WBC) count, lymphocyte count, monocyte count, hemoglobin, hematocrit, platelet count, serum high-density lipoprotein cholesterol (HDLC), serum low-density lipoprotein cholesterol (LDLC), serum alkaline phosphatase (ALP), serum total bilirubin (TBIL), serum gamma-glutamyl transferase (GGT), serum alanine transaminase (ALT), serum aspartate transaminase (AST), serum total protein (TP), serum osmolality, serum lactate dehydrogenase (LDH), serum globulin, serum apolipoprotein B (apoB), serum apolipoprotein AI (apoAI), serum total cholesterol (TC), serum triglycerides (TG), plasma fibrinogen, urinary albumin, ferritin, and serum C-reactive protein (CRP).

### External validation

This study utilized national health and nutrition examination survey (NHANES) data spanning from January 1988 to December 2018 for external validation. The NHANES protocol, which was approved by the National Center for Health Statistics Research Ethics Review Board, adhered to rigorous ethical standards, and all participants provided written informed consent. A total of 1559 patients with DKD were included in the external validation dataset. Patients were categorized into 979 cases in the early group (G1–G2) and 580 cases in the advanced group (G3–G5). The inclusion criteria and the collected variables for all patients were consistent with those of the internal dataset.

The NHANES was chosen as the external validation set for four key methodological and clinical reasons: (1) Heterogeneous population verification: The internal multicenter dataset originated from tertiary hospitals in Eastern China (predominantly Han Chinese), whereas NHANES is a nationally representative cross-sectional survey in the United States, including diverse ethnic groups (Caucasian, African American, Hispanic, etc.), different healthcare systems, and varying lifestyle characteristics. This cross-ethnic, cross-regional, cross-healthcare system validation is the gold standard for evaluating the generalizability of prediction models and effectively assesses the applicability of the model to diverse DKD patient populations globally. (2) Standardized and high-quality data: NHANES uses standardized laboratory testing methods, strict quality control, and detailed clinical variable collection, thereby ensuring the reliability of the external validation data. (3) Complementary clinical scenario: The internal dataset included patients with confirmed DKD who were referred to tertiary hospitals (severe disease bias), whereas NHANES includes community-dwelling individuals with DKD (mild to moderate disease). This complementary scenario allows us to verify the model’s performance in both clinical and community settings, which is essential for its utility in early screening. (4) Public availability and reproducibility: NHANES data are publicly available, allowing other researchers to reproduce our model and verify the results independently, thereby enhancing the study’s transparency and scientific rigor.

### Data preprocessing

Missing data in the internal dataset and the NHANES external dataset were imputed independently using the missForest algorithm at the individual dataset level, without the prior combination of the two datasets before imputation. missForest outperforms established imputation methods, such as k-nearest neighbor imputation and multivariate imputation using chained equations. MissForest can simultaneously handle multivariate data consisting of both continuous and categorical variables, without requiring parameter tuning (15). By imputing the missing data, missForest maintains the integrity of the dataset and ensures the reliability of subsequent analyses.

### Selection of variables

The combination of LASSO and RFE will be employed to select the quantitative variables: (1) LASSO will use 10-fold cross-validation (CV) to select the optimal λ value, minimizing the cross-validation error. This process will compress the coefficients of non-essential variables to zero, yielding an initial variable set with significant predictive value. (2) RFE will be applied to this initial variable set, reducing the number of variables from the maximum down to one based on the LR model. The optimal variable set will be determined by the accuracy curve from RFE, selecting the number of variables that results in the maximum model accuracy.

### Model development and validation

The internal dataset was randomly divided into a training set and a test set in a 7:3 ratio. Eight machine learning models, including LASSO, RF, XGB, logistic regression (LR), light gradient boosting machine (LGBM), gradient boosting machine (GBM), support vector machine (SVM), and naive bayes (NB), were used to predict the occurrence of advanced DKD. Hyperparameter optimization was performed on all eight ML models to ensure optimal model performance. The grid search method was used for hyperparameter tuning, and the performance of different hyperparameter combinations was evaluated by 10-fold CV on the internal training set. For each model, the optimal hyperparameter set was selected based on the highest AUC value (the primary evaluation measure).

The performance of the prediction model was evaluated using the area under the receiver operating characteristic curve (AUC), accuracy, sensitivity, specificity, positive predictive value (PPV), NPV, F1 score, and Brier score. Additionally, calibration curves were plotted to assess the accuracy of the model’s predicted probabilities, and decision curve analysis (DCA) was performed to evaluate the net benefit of the model across different decision thresholds. The model was developed based on the training set, after which the prediction model with superior performance—according to the aforementioned evaluation metrics—was selected and subsequently validated on the test set and external datasets.

### Model explanation

SHAP is employed to interpret the prediction model. SHAP is a model interpretation method grounded in game theory. It provides both local and global explanations by calculating the average contribution of each variable to the model’s predictions, thus addressing the “black box” problem and enhancing the model’s transparency and interpretability. Moreover, the PDP visually illustrates the marginal effect of a single variable on the model’s predictions, aiding in the identification of complex nonlinear relationships between variables and prediction outcomes, thereby enhancing the transparency of the model’s decision-making process. SHAP and PDP analyses were performed using the R programming language (version 4.5.0), calculated and visualized using the shapviz (version 0.9.0) and PDP (version 0.8.1) packages.

### Network calculator

To facilitate the application of the model in a clinical setting, the final prediction model was integrated into a network platform based on the Shiny application. When the values of the relevant variables in the final model are provided, the application returns the probability of occurrence, identifies important features, and generates bee colony maps and waterfall maps for the advanced DKD.

### Determination of minimum sample size

The sample size required for this study was determined based on the clinical prediction model sample size calculation method proposed by Riley et al. (16). The first step involves determining the number of samples needed to accurately estimate the average risk or mean. The second step involves determining the number of samples required for the error between the predicted and true values of the model to be minimized. The third step involved determining whether there was an adequate sample size to prevent overfitting. The fourth step involves determining whether there is a sufficient sample size to minimize the error between the predicted and actual performance of the model. Finally, the maximum value from the results of the above four-step calculation was selected as the required sample size.

### Statistics

Statistical analyses were performed using R (version 4.5.0, R Foundation). Continuous variables with a normal distribution are presented as the mean ± standard deviation and were compared using the t-test. Continuous variables with skewed distributions are presented as medians with interquartile ranges and compared using the Mann–Whitney U test or the Kruskal–Wallis H test. Categorical variables are presented as counts with percentages and compared using the chi-square test. The AUC was used to evaluate predictive power, and the optimal cutoff value was determined by maximizing the Youden index. A two-tailed P value < 0.05 was considered statistically significant.

## Results

### Clinical characteristics

**Table 1** presents the demographic and clinical characteristics of all patients in the internal and external datasets. In the internal dataset, compared with patients in the early stage of DKD, those in the advanced stage of DKD exhibited a higher incidence of hypertension, anemia, and heart failure. Additionally, patients with advanced DKD were older and had a lower BMI. The assessment of laboratory characteristics revealed that serum urea, serum UA, serum IP, serum creatinine, neutrophil count, serum ALP, serum osmolality, serum LDH, and serum globulin levels were all higher in patients with advanced DKD. In contrast, these patients exhibited decreased levels of serum calcium, serum albumin, serum bicarbonate, lymphocyte count, hemoglobin, hematocrit, platelet count, serum HDLC, serum LDLC, serum TBIL, serum ALT, serum TP, serum apoB, serum apoAI, serum TC, and serum ferritin compared to patients with early DKD.

**Table 1:** The comparison of the demographic and clinical characteristics of patients with early DKD and advanced DKD in both internal and external datasets.

Variable	Early DKD (internal dataset) n = 223	Advanced DKD (internal dataset) n = 2136	P value	Early DKD (external dataset) n = 979	Advanced DKD (external dataset) n = 580	P value
Male, n(%)	140 (62.8%)	1448 (67.8%)	0.149	511 (52.2%)	251 (43.3%)	**<0.001**
Alcohol consumption history, n(%)	20 (17.7%)	171 (14.7%)	0.468	431 (44.0%)	188 (32.4%)	**<0.001**
Smoking history, n(%)	40 (34.8%)	351 (29.7%)	0.301	107 (10.9%)	77 (13.3%)	0.191
Hypertension, n(%)	185 (83.0%)	2010 (94.1%)	**<0.001**	521 (53.7%)	441 (77.4%)	**<0.001**
Anemia, n(%)	37 (16.6%)	1543 (72.2%)	**<0.001**	26 (2.7%)	44 (7.7%)	**<0.001**
Heart failure, n(%)	13 (5.8%)	659 (30.9%)	**<0.001**	59 (6.0%)	82 (14.1%)	**<0.001**
Malignancy, n(%)	21 (9.4%)	114 (5.3%)	**0.019**	68 (6.9%)	109 (18.8%)	**<0.001**
Age, years	63.00 (56.50–71.00)	68.00 (59.00–77.00)	**<0.001**	56.00 (46.00–65.00)	70.00 (63.00–76.00)	**<0.001**
BMI, kg/m^2^	24.64 (22.88–26.86)	24.09 (21.97–26.37)	**0.022**	28.25 (24.90–32.20)	27.70 (24.54–31.28)	0.059
Serum urea, mmol/L	6.25 (4.90–7.81)	16.27 (9.98–24.45)	**<0.001**	5.00 (3.93–6.07)	6.78 (5.71–8.57)	**<0.001**
Serum UA, umol/L	371.00 (290.00–444.00)	438.00 (357.25–528.00)	**<0.001**	309.30 (249.80–368.80)	350.90 (291.50–417.87)	**<0.001**
Serum IP, mmol/L	1.16 (1.02–1.26)	1.38 (1.13–1.77)	**<0.001**	1.13 (1.00–1.23)	1.10 (1.00–1.25)	0.535
Serum creatinine, umol/L	84.00 (66.00–107.75)	312.00 (155.00–611.00)	**<0.001**	88.40 (79.56–97.24)	114.92 (97.24–132.60)	**<0.001**
Serum calcium, mmol/L	2.28 (2.17–2.36)	2.15 (2.00–2.29)	**<0.001**	1.23 (1.20–1.26)	1.23 (1.20–1.26)	0.342
Serum albumin, g/L	40.20 (34.00–43.20)	34.00 (29.00–38.70)	**<0.001**	42.00 (40.00–44.00)	41.00 (39.00–44.00)	**<0.001**
Serum bicarbonate, mmol/L	25.00 (23.00–27.00)	23.00 (20.00–25.80)	**<0.001**	29.00 (26.00–32.00)	29.00 (26.00–32.00)	0.163
Serum glucose, mmol/L	7.10 (5.68–8.95)	6.78 (5.26–9.38)	0.369	8.14 (7.28–10.25)	8.21 (7.20–10.60)	0.807
Neutrophil count, 1000 cells/uL	4.30 (3.32–5.57)	4.70 (3.70–6.43)	**0.007**	4.44 (3.55–5.50)	4.70 (3.65–5.84)	**0.024**
WBC, 1000 cells/uL	7.00 (5.75–8.26)	6.97 (5.70–8.73)	0.499	7.30 (6.05–8.60)	7.31 (6.05–8.85)	0.344
Lymphocyte count, 1000 cells/uL	1.73 (1.37–2.19)	1.33 (0.95–1.80)	**<0.001**	2.20 (1.80–2.75)	2.15 (1.70–2.70)	**0.013**
Monocyte count, 1000 cells/uL	0.46 (0.35–0.58)	0.45 (0.35–0.60)	0.982	0.40 (0.30–0.50)	0.40 (0.30–0.55)	0.083
Hemoglobin, g/L	133.00 (118.50–147.00)	103.00 (85.00–123.00)	**<0.001**	140.41 ± 14.87	135.82 ± 15.40	**<0.001**
Hematocrit	0.40 (0.36–0.44)	0.31 (0.26–0.37)	**<0.001**	0.42 ± 0.04	0.41 ± 0.04	**<0.001**
Platelet count, 1000 cells/uL	218.00 (179.50–276.50)	206.00 (163.00–254.00)	**0.004**	276.00 (232.00–331.12)	267.50 (225.12–318.25)	**0.017**
Serum HDLC, mmol/L	0.99 (0.86–1.27)	0.94 (0.76–1.17)	**<0.001**	1.21 (1.01–1.47)	1.22 (1.01–1.50)	0.556
Serum LDLC, mmol/L	3.07 (2.09–3.66)	2.41 (1.79–3.26)	**<0.001**	3.44 (2.84–4.03)	3.59 (2.92–4.29)	0.092
Serum ALP, U/L	68.00 (58.00–82.00)	79.00 (61.00–104.00)	**<0.001**	87.00 (73.00–104.00)	89.00 (73.00–107.75)	0.229
Serum TBIL, umol/L	8.45 (5.90–11.15)	6.45 (4.50–9.40)	**<0.001**	8.55 (6.84–11.97)	8.55 (6.84–10.48)	**0.013**
Serum GGT, U/L	26.20 (19.00–46.50)	29.00 (19.00–53.00)	0.343	29.50 (19.00–45.00)	23.25 (17.00–36.00)	**<0.001**
Serum ALT, U/L	18.00 (13.75–27.25)	14.00 (10.00–23.00)	**<0.001**	15.00 (11.00–22.00)	12.00 (9.00–17.00)	**<0.001**
Serum AST, U/L	19.00 (15.50–24.00)	18.00 (14.00–25.00)	0.308	20.00 (16.75–26.00)	19.00 (16.00–23.00)	**0.011**
Serum TP, g/L	65.70 (57.00–72.00)	62.00 (55.20–69.00)	**0.002**	74.00 (71.00–77.00)	74.00 (70.88–77.00)	0.152
Serum osmolality, mmol/Kg	282.51 (277.62–287.08)	289.84 (282.24–298.10)	**<0.001**	275.00 (271.00–278.00)	279.00 (274.00–283.00)	**<0.001**
Serum LDH, U/L	189.00 (161.00–219.75)	215.00 (176.00–270.45)	**<0.001**	149.00 (131.00–169.00)	160.00 (139.75–180.00)	**<0.001**
Serum globulin, g/L	26.15 (23.00–30.00)	28.00 (24.00–32.00)	**0.013**	32.00 (29.00–35.00)	32.00 (29.00–36.00)	0.635
Serum apoB, g/L	1.01 (0.78–1.23)	0.84 (0.66–1.07)	**<0.001**	1.12 (0.97–1.29)	1.18 (1.00–1.38)	**<0.001**
Serum apoAI, g/L	1.21 (1.08–1.38)	1.09 (0.89–1.27)	**<0.001**	1.40 (1.26–1.57)	1.44 (1.27–1.60)	0.073
Serum TC, mmol/L	4.64 (3.79–5.47)	4.00 (3.21–4.99)	**<0.001**	5.64 (4.94–6.37)	5.92 (5.12–6.72)	**<0.001**
Serum TG, mmol/L	1.54 (1.06–2.12)	1.48 (1.05–2.19)	0.498	1.61 (1.16–2.37)	1.84 (1.24–2.69)	**0.001**
Plasma fibrinogen, g/L	3.60 (2.85–4.66)	4.02 (3.20–4.94)	**0.003**	3.02 (2.58–3.60)	3.34 (2.85–4.07)	**<0.001**
Urinary albumin, mg/L	315.00 (18.25–1585.00)	845.00 (143.75–2215.00)	**0.002**	9.40 (3.82–22.00)	13.60 (5.00–49.00)	**<0.001**
Serum ferritin, ug/L	680.54 (430.40–716.80)	214.70 (99.00–431.10)	**0.024**	132.00 (62.25–243.00)	127.00 (68.00–236.50)	0.773
Serum CRP, mg/L	4.90 (1.40–26.70)	12.00 (2.84–46.05)	**0.048**	0.21 (0.21–0.66)	0.21 (0.21–0.77)	0.195

DKD, diabetic kidney disease; BMI, body mass index; Serum UA, serum uric acid; Serum IP, serum inorganic phosphorus; WBC, white blood cell count; Serum HDLC, serum high-density lipoprotein cholesterol; Serum LDLC, serum low-density lipoprotein cholesterol; Serum ALP, serum alkaline phosphatase; Serum TBIL, serum total bilirubin; Serum GGT, serum gamma glutamyl transferase; Serum ALT, serum alanine aminotransferase; Serum AST, serum aspartate aminotransferase; Serum TP, serum total protein; Serum LDH, serum lactate dehydrogenase; Serum apoB, serum apolipoprotein B; Serum apoAI, serum apolipoprotein AI; Serum TC, serum total cholesterol; Serum TG, serum triglycerides; Serum CRP, serum C-reactive protein. Bold values indicate statistically significant differences.

In the external dataset, the incidences of hypertension, anemia, heart failure, and malignant tumors in patients with advanced DKD were significantly higher than those in patients with early DKD, and the age of patients with advanced DKD was also greater. The laboratory characteristic assessment revealed that, compared with patients with early DKD, patients with advanced DKD exhibited elevated levels of serum urea, serum UA, serum creatinine, neutrophil count, serum osmolality, serum LDH, serum apoB, serum TC, serum TG, plasma fibrinogen, and urinary albumin. In contrast, serum albumin, lymphocyte count, hemoglobin, hematocrit, platelet count, serum TBIL, serum GGT, serum ALT, and serum AST levels decreased significantly in patients with advanced DKD.

### Selection of variables

**Figure 2:**
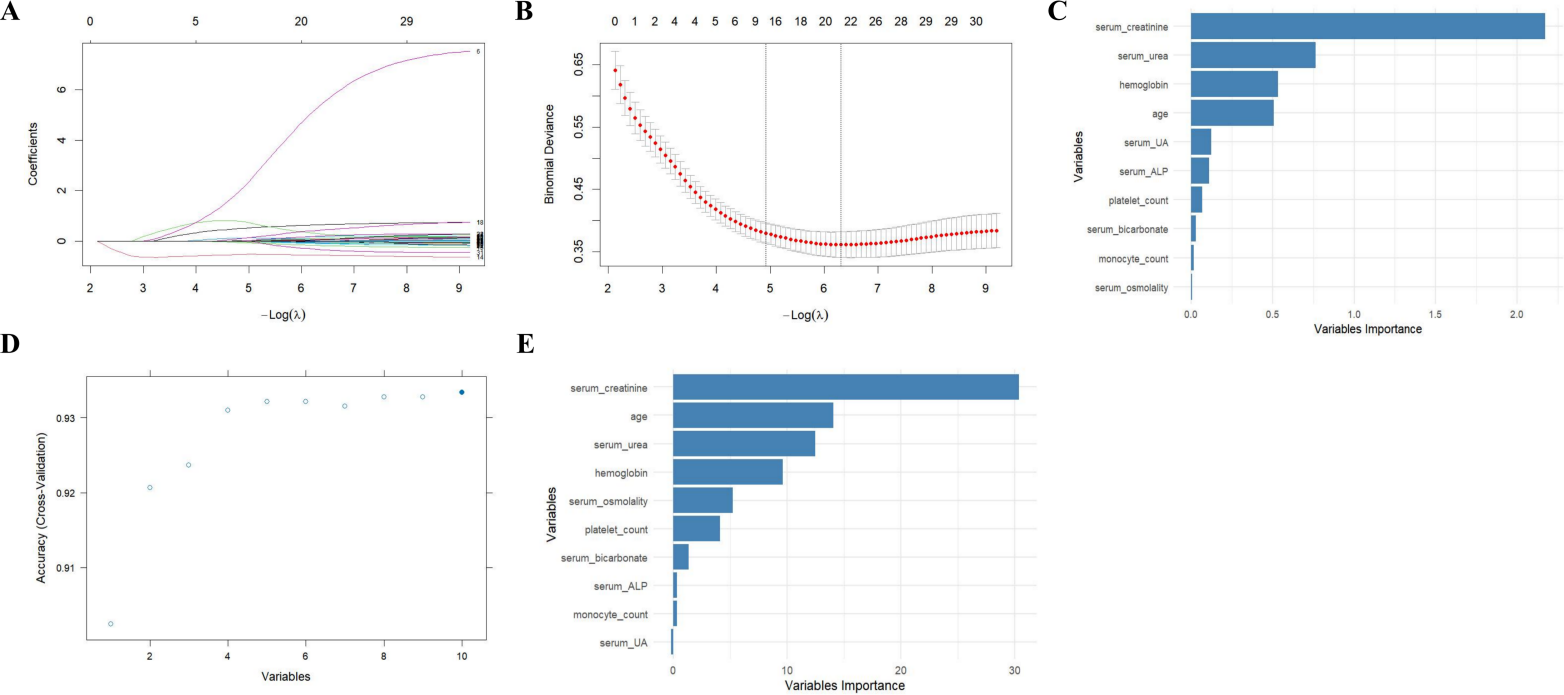
LASSO and RFE were combined for variable screening. **(A)** The LASSO coefficient path; **(B)** The LASSO cross-validation error curves; **(C)** The absolute importance of variables obtained through LASSO screening; **(D)** The relationship between the number of variables in RFE and the accuracy; **(E)** The absolute importance of variables obtained through RFE screening.

As shown in **Figures 2A–C**, the LASSO CV curve indicates that the optimal λ value, corresponding to the minimum CV error, occurs when the number of variables is 10. This identifies the top 10 variables as the primary screening variable set, which exhibits significant predictive value. Based on the LR model, RFE was performed on this primary screening variable set. The RFE accuracy curve demonstrated that when the number of variables was 10, the model’s accuracy reached its maximum value and remained stable. In contrast, when the number of variables was reduced to fewer than 10, the model’s accuracy decreased (**Figure 2D**). Ultimately, the optimal variable set consists of: serum creatinine, age, serum urea, hemoglobin, serum osmolality, platelet count, serum bicarbonate, serum ALP, monocyte count, and serum UA (**Figure 2E**).

### Model development and validation

**Figure 3:**
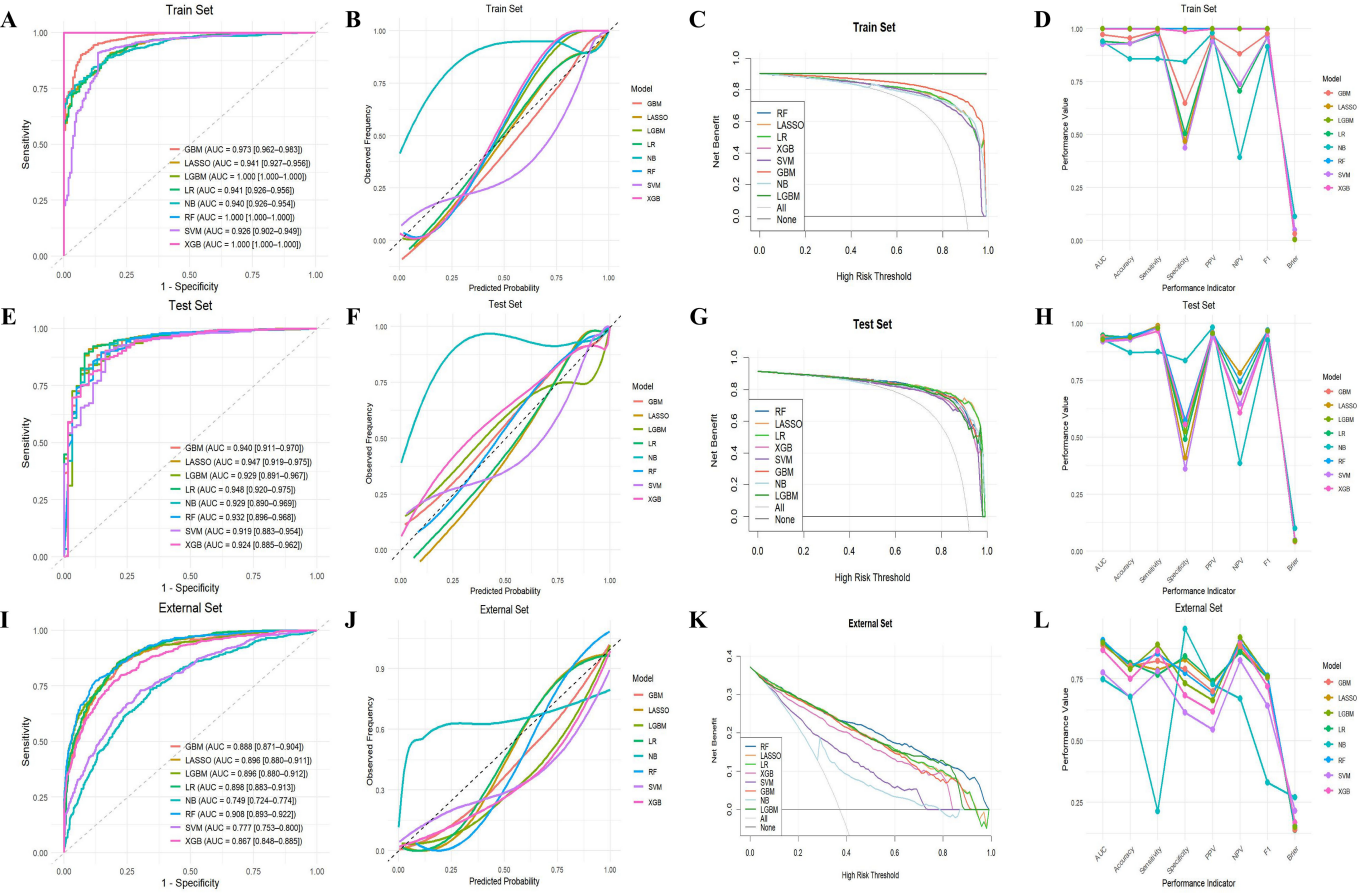
The performance of ML models for predicting the occurrence of DKD in the train, test, and external sets. ROC curve analysis **(A, E, I)**, calibration curve analysis **(B, F, J)**, DCA curves **(C, G, K)**, and parallel line graphs of performance indicators for each model **(D, H, L)** were used to predict the occurrence of DKD in the train, test, and external sets using eight ML algorithms. ML, machine learning; DKD, diabetic kidney disease; ROC, receiver operating characteristic; DCA, decision curve analysis; GBM, gradient boosting machine; LASSO, least absolute shrinkage and selection operator; LGBM, light gradient boosting machine; LR, logistic regression; NB, naive bayes; RF, random forest; SVM, support vector machine; XGB, extreme gradient boosting.

On the basis of the training set, eight ML models were constructed using the optimal set of variables and further validated using the test set for internal validation and the NHANES dataset for external validation. As shown in **Figures 3A, C**, in the training set, the LR model showed excellent performance in terms of discrimination and net benefit, with an AUC of 0.941 (95% CI: 0.926-0.956) and net benefit within the full threshold range of 0-1.0. In addition to good calibration (Brier score = 0.05), the LR model also had high accuracy (0.931), sensitivity (0.977), PPV (0.948), NPV (0.707), and F1 score (0.962) (**Figures 3B, D**; **Table 2**).

**Figure 4:**
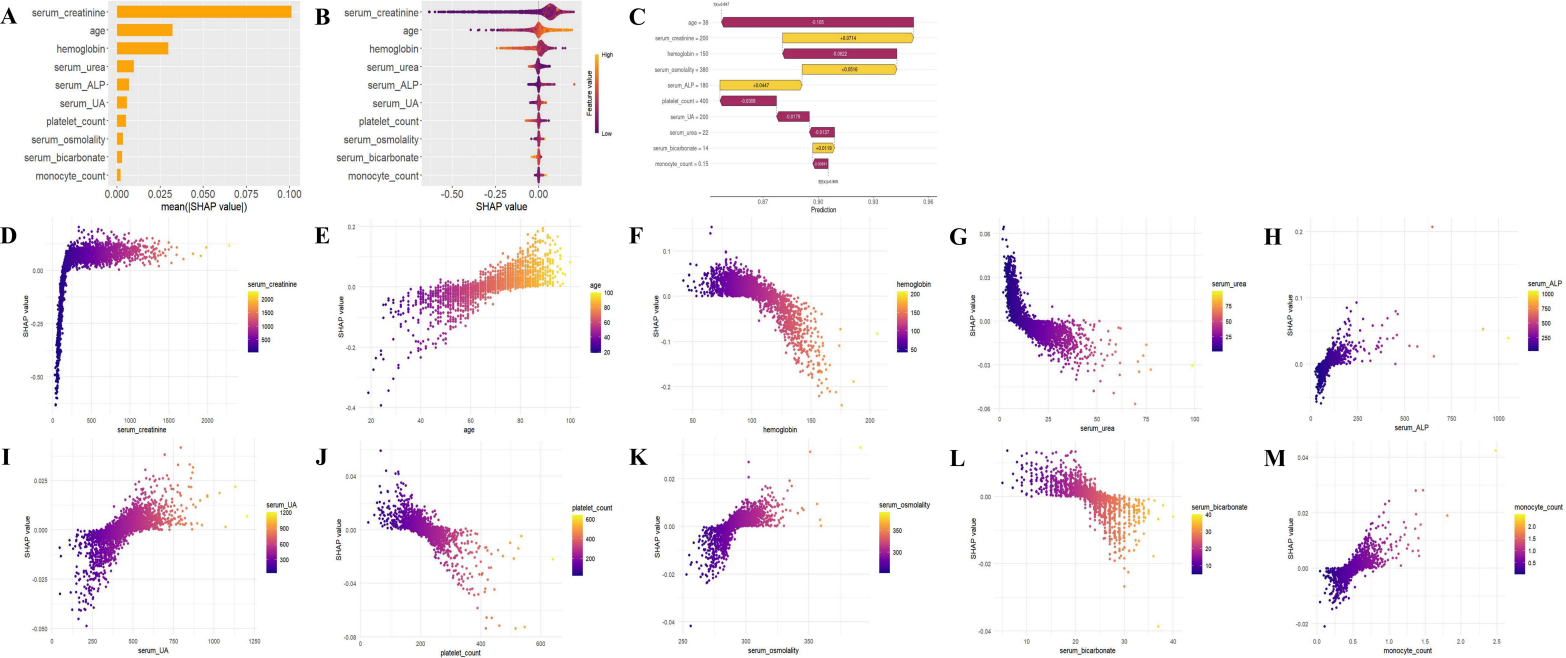
The model is interpreted both globally and locally using SHAP. **(A)** SHAP variable importance bar plot. This plot evaluates the contribution of each variable to the model based on the mean SHAP values, which are displayed in descending order. **(B)** SHAP beeswarm plot: The probability of advanced CKD increases with the SHAP values of the variables. Each dot represents a patient’s SHAP value for a given variable, where orange denotes higher variable values and purple denotes lower values. The dots are stacked vertically to display the density. **(C)** SHAP waterfall plot: This plot illustrates the contribution of each variable to the patient’s prediction result using the LR model. Orange bars indicate variables contributing positively to the prediction, while purple bars indicate negative contributions. Variable values are presented alongside their SHAP values, highlighting key variables such as age (-0.105), serum creatinine (+0.0714), hemoglobin (-0.0622), serum osmolality (+0.0516), serum ALP (+0.0447), platelet count (-0.0308), serum UA (-0.0179), serum urea (-0.0137), serum bicarbonate (+0.0119), and monocyte count (-0.00841). The overall contribution is 0.905, with a baseline contribution of 0.847. **(D–M)** SHAP dependence plot: Each dependence plot illustrates how a single variable influences the model’s output, with each point representing a patient. SHAP: SHapley Additive exPlanations; DKD: diabetic kidney disease; LR: logistic regression.

**Table 2:** The performance parameters of the eight machine learning prediction models, evaluated in the training set, test set, and external set.

Model	Set	AUC	Accuracy	Sensitivity	Specificity	PPV	NPV	F1	Brier
RF	Train	1	1	1	1	1	1	1	0.007
LGB	Train	1	1	1	1	1	1	1	0.003
XGB	Train	1	0.999	1	0.988	0.999	1	0.999	0.007
GBM	Train	0.973	0.957	0.991	0.648	0.963	0.882	0.976	0.032
LASSO	Train	0.941	0.932	0.982	0.469	0.944	0.738	0.963	0.051
LR	Train	0.941	0.931	0.977	0.506	0.948	0.707	0.962	0.05
NB	Train	0.94	0.857	0.858	0.846	0.981	0.394	0.915	0.112
SVM	Train	0.926	0.93	0.983	0.438	0.941	0.74	0.962	0.051
LR	Test	0.948	0.938	0.98	0.492	0.953	0.698	0.966	0.042
LASSO	Test	0.947	0.939	0.989	0.41	0.947	0.781	0.967	0.043
GBM	Test	0.94	0.939	0.978	0.525	0.956	0.696	0.967	0.043
RF	Test	0.932	0.946	0.981	0.574	0.961	0.745	0.971	0.044
NB	Test	0.929	0.871	0.875	0.836	0.983	0.386	0.926	0.101
LGB	Test	0.929	0.939	0.978	0.525	0.956	0.696	0.967	0.046
XGB	Test	0.924	0.931	0.966	0.557	0.959	0.607	0.962	0.049
SVM	Test	0.919	0.928	0.981	0.361	0.942	0.647	0.961	0.05
RF	External	0.908	0.804	0.853	0.774	0.691	0.899	0.764	0.154
LR	External	0.898	0.814	0.767	0.842	0.742	0.859	0.754	0.139
LGB	External	0.896	0.791	0.89	0.732	0.663	0.918	0.76	0.151
LASSO	External	0.896	0.814	0.788	0.829	0.732	0.868	0.759	0.144
GBM	External	0.888	0.803	0.824	0.791	0.7	0.884	0.757	0.139
XGB	External	0.867	0.75	0.864	0.683	0.618	0.894	0.72	0.169
SVM	External	0.777	0.676	0.781	0.614	0.545	0.826	0.642	0.217
NB	External	0.749	0.678	0.214	0.953	0.729	0.672	0.331	0.27

RF, random forest; LGBM, light gradient boosting machine; XGB, extreme gradient boosting; GBM, gradient boosting machine; LR, logistic regression; LASSO, least absolute shrinkage and selection operator; NB, naive bayes; SVM, support vector machine; AUC, area under the receiver operating characteristic curve; PPV, positive predictive value; NPV, negative predictive value; F1, F1 score; Brier, Brier score.

The validation results of the test set showed that the AUC of the LR model was higher than that of other ML models, reaching 0.948 (95%CI: 0.920-0.975), which effectively distinguished the occurrence of DKD (**Figure 3E**). The LR model performed better calibration than all models (Brier score = 0.042) with a net benefit across the entire threshold range (**Figures 3F, G**; **Table 2**). In **Figure 3H** and **Table 2**, the LR model remained robust for accuracy (0.938), sensitivity (0.98), PPV (0.953), NPV (0.698), and F1 score (0.966). This suggests that the LR model maintains stable performance in internal validation as a screening tool for advanced DKD.

**Figure 3I, K** show that the LR model showed strong discrimination and net benefit in the external validation, with an AUC of 0.898 (95%CI: 0.883-0.913) and net benefit coverage close to 1. In addition, as shown in **Figure 3J** and **Table 2**, the calibration of the LR model was better than that of the other models (Brier score = 0.139). Notably, the LR model not only achieved the highest accuracy (0.814) and PPV (0.742), but also showed strong performance in terms of sensitivity (0.767), specificity (0.842), NPV (0.859), and F1 score (0.754) (**Figures 3L**; **Table 2**). The results of this study suggest that the LR model has good applicability in external validation and can be used as an effective screening tool for advanced DKD.

### Model explanation

Given that prediction models that are entirely inexplicable or lack direct interpretability are generally not accepted by clinicians, this study employs the SHAP method to assess the contribution of each variable to the prediction, aiming to elucidate the output of the final model. This method provides both a global interpretation at the feature level and a local interpretation at the individual level. More specifically, the global interpretation describes the overall functionality of the model. As illustrated in **Figures 4A**, the SHAP variable importance bar plot displays the average absolute SHAP value for each variable and its specific contribution to the model. Serum creatinine, age, hemoglobin, serum urea, serum ALP, serum UA, platelet count, serum osmolality, serum bicarbonate, and monocyte count rank as the ten most important variables in the prediction model, in that order. The SHAP beeswarm plot visualizes the direction and intensity of each variable’s influence on the model’s prediction. For instance, elevated serum creatinine, age, serum ALP, serum UA, serum osmolality, and monocyte count, combined with low hemoglobin, serum urea, platelet count, and serum bicarbonate, were identified as significant risk factors for advanced DKD (**Figure 4B**). The SHAP dependence plot in **Figures 4D–M** aids in understanding the specific impact of each variable on the output of the predictive model by comparing the actual values of the variables with their corresponding SHAP values. For instance, the actual values of the variables corresponding to SHAP values above zero have a positive predictive effect on the model, indicating a higher risk of advanced DKD.

In addition, the SHAP waterfall plot illustrates both the direction and magnitude of each variable’s contribution to the prediction results of a single sample, alongside the sum of all cumulative contributions, offering a local interpretation of the model at the individual level. As depicted in **Figure 4C**, the serum creatinine value of 200, serum osmolality value of 380, serum ALP value of 180, and serum bicarbonate value of 14 all made significant positive contributions to the prediction results (+0.0714, +0.0516, +0.0447, and +0.0119, respectively). In contrast, the age value of 38, hemoglobin value of 150, platelet count value of 400, serum UA value of 200, serum urea value of 22, and monocyte count value of 0.15 all made significant negative contributions to the prediction results (-0.105, -0.0622, -0.0308, -0.0179, -0.0137, and -0.00841, respectively). Therefore, the SHAP waterfall plot visually demonstrates the cumulative influence paths of each variable on the prediction results of individual patients, assisting clinicians in gaining a deeper understanding of the model’s decision-making mechanism, thereby enabling the formulation of more precise treatment plans.

**Figure 5:**
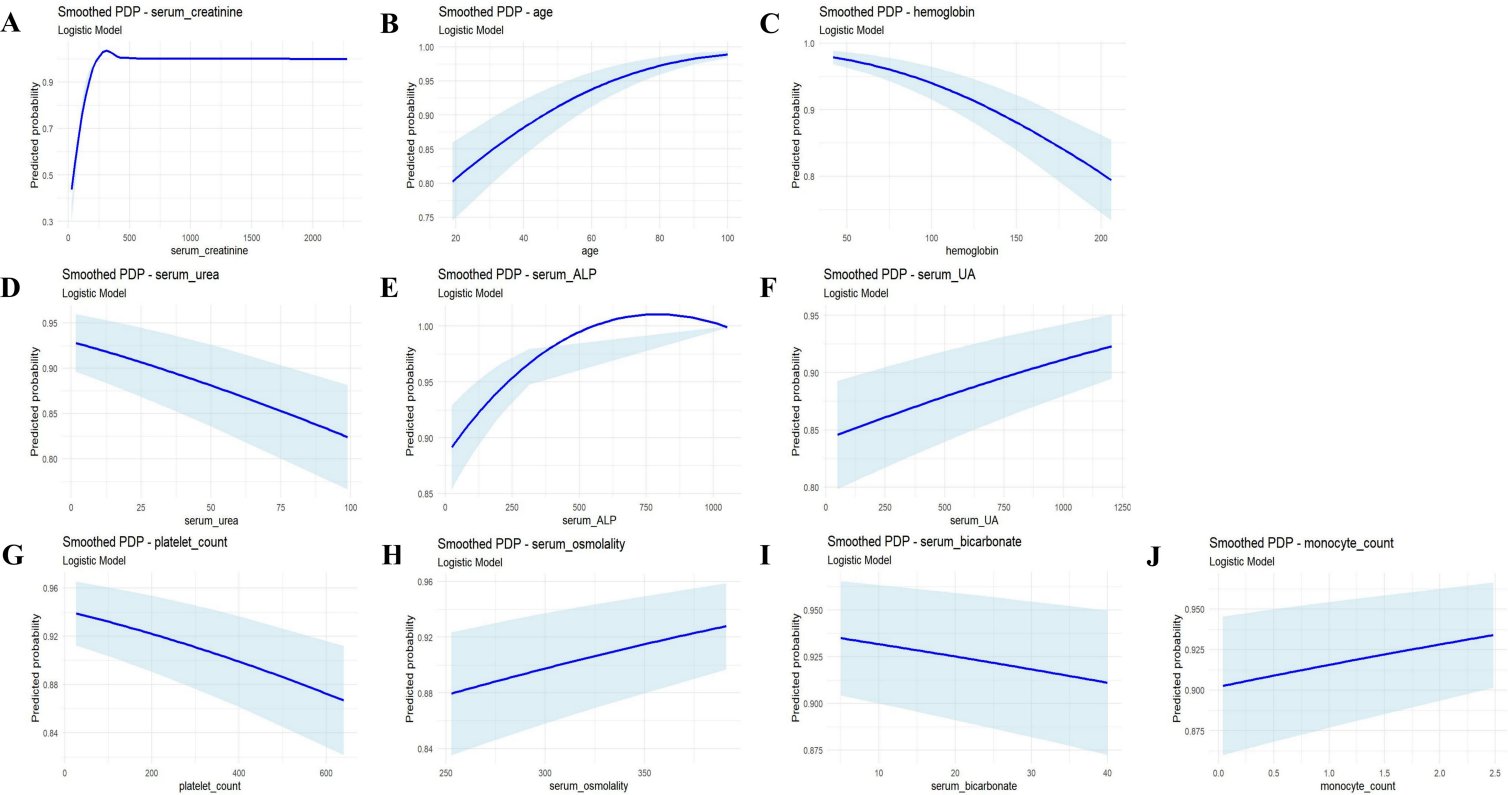
PDP from the LR model for predicting advanced DKD, showing the marginal effects of serum creatinine, age, hemoglobin, serum urea, serum ALP, serum UA, platelet count, serum osmolality, serum bicarbonate, and monocyte count **(A-J)**. PDP, Partial Dependence Plot; LR, logistic regression; DKD, diabetic kidney disease.

PDP analysis further elucidated the marginal effect of key variables on the model’s predictions. As shown in **Figure 5**, serum creatinine, age, serum ALP, serum UA, serum osmolality and monocyte count were positively correlated with the development of advanced DKD, whereas hemoglobin, serum urea, platelet count and serum bicarbonate exhibited a negative correlation.

### Application of the web calculator

**Figure 6:**
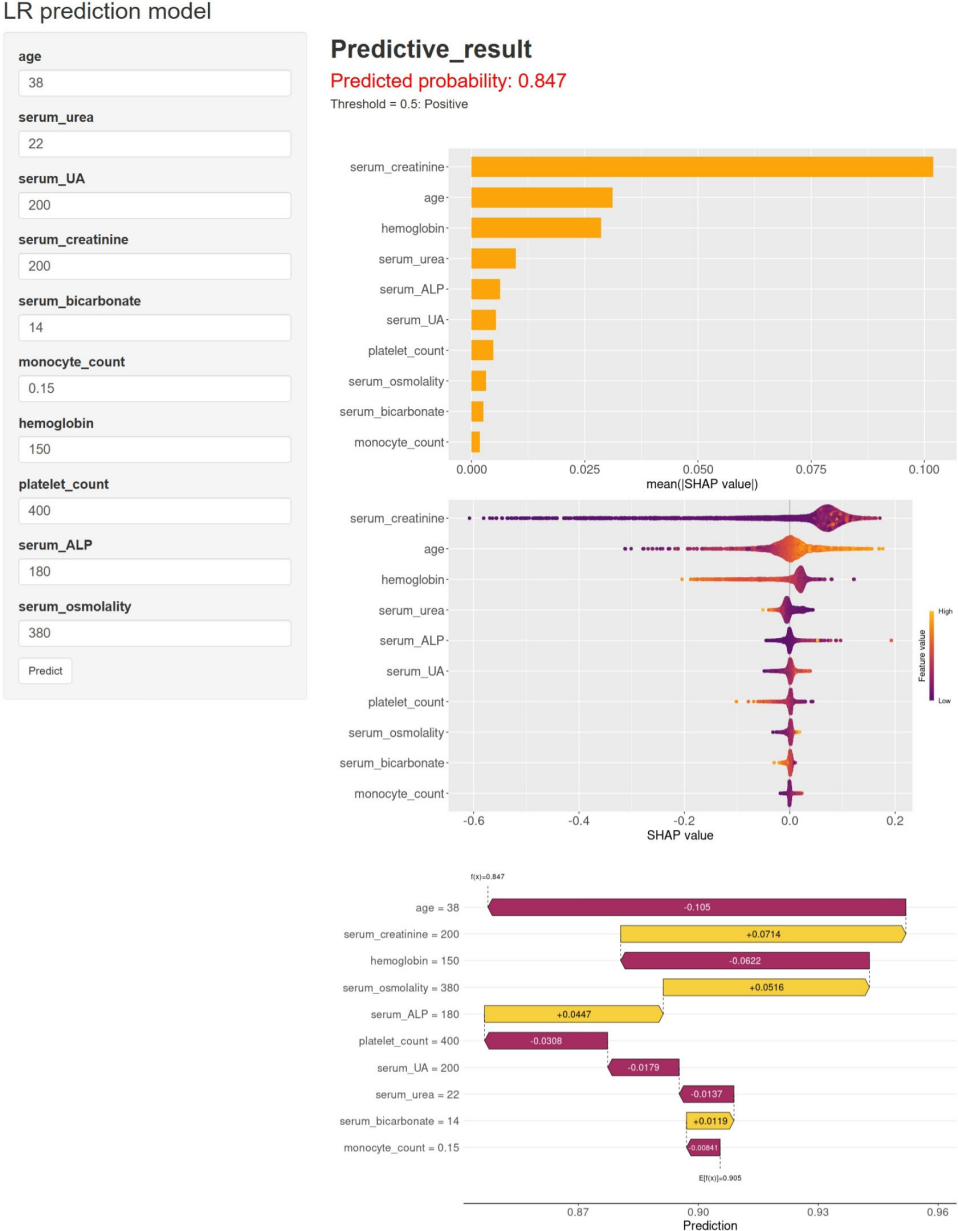
Web-based models for predicting the occurrence of advanced DKD. The risk of advanced DKD occurrence can be predicted by simply inputting the values of age, serum urea, serum UA, serum creatinine, serum bicarbonate, monocyte count, hemoglobin, platelet count, serum ALP, and serum osmolality. DKD, diabetic kidney disease; LR, logistic regression.

The final prediction model has been integrated into a web application for direct use by clinicians, as illustrated in **Figure 6**. By inputting the actual values of age, serum urea, serum UA, serum creatinine, serum bicarbonate, monocyte count, hemoglobin, platelet count, serum ALP, and serum osmolality, the application automatically predicts the risk of advanced DKD occurrence. The web application is accessible online through the following link: https://dev2333.shinyapps.io/logistics1/.

## Discussion

Most current clinical prediction models for DKD are based on single-center studies. Even in multi-center studies, the data often reflect distinct regional characteristics, which significantly limits the potential for broader application across different centers or regions (17–19). This study developed a model to predict the occurrence of advanced DKD, utilizing the EMR from Fuzhou University Affiliated Provincial Hospital in Fujian, China, and externally validated it using the NHANES database from the United States. This approach greatly enhances the generalizability of the model, facilitating its broader adoption in clinical practice. However, the study observed notable differences between DKD patients in the internal dataset from Fuzhou University Affiliated Provincial Hospital and the external NHANES dataset. These differences were observed in various demographic and laboratory variables, including gender, alcohol consumption history, malignancy, BMI, serum IP, serum calcium, serum bicarbonate, platelet count, serum HDLC, serum LDLC, serum ALP, serum GGT, serum AST, serum TP, serum globulin, serum apoB, serum apoAI, serum TC, serum TG, serum ferritin, and serum CRP. These differences can be attributed to variations in genetic susceptibility, environmental factors, lifestyle, and dietary habits between the two populations, as the datasets originate from distinct regions (20–23). Furthermore, while the external dataset is derived from population surveys, the internal dataset originates from inpatient EMRs, which tend to reflect more complex conditions that are influenced by various external factors. As a result, there are inherent differences in the demographic and laboratory characteristics between the two datasets.

In this study, variable selection was performed using a combination of the LASSO and RFE methods, which identified an optimal set of ten variables: serum creatinine, age, hemoglobin, serum urea, serum ALP, serum UA, platelet count, serum osmolality, serum bicarbonate, and monocyte count. Subsequently, eight established ML models were constructed and validated based on this refined variable set. Our result revealed that the LR model demonstrated strong performance in the test set and achieved the best discrimination (AUC = 0.948) and net benefit across the entire decision threshold range upon internal validation. Furthermore, the LR model remained robust for accuracy (0.938), sensitivity (0.98), PPV (0.953), NPV (0.698), and F1 score (0.966), indicating its robustness as a screening tool for advanced DKD. In external validation, the LR model maintained the strongest discriminatory power (AUC = 0.898) compared to other models, along with the greatest accuracy (0.814) and PPV (0.742). These results indicate that the LR model generalizes well to external cohorts and represents an effective screening tool for advanced DKD.

In the SHAP analysis, serum creatinine, age, hemoglobin, serum urea, serum ALP, serum UA, platelet count, serum osmolality, serum bicarbonate, and monocyte count were identified as the most important variables in the advanced DKD model, which aligns with the pathophysiological mechanisms of DKD. Almost all studies have demonstrated that serum creatinine and serum urea are highly correlated with the occurrence and progression of advanced DKD (24, 25). Furthermore, Yamanouchi et al. reported that the level of hemoglobin was negatively correlated with renal pathological features, particularly the severity of interstitial fibrosis, and could predict the progression of DKD (26). In line with this, our study found that hemoglobin levels were negatively correlated with the risk of advanced DKD. Our research also confirmed that age and serum osmolality were positively correlated with the risk of developing advanced DKD. This finding aligns with previous studies identifying advanced age and high serum osmolality as independent risk factors for adverse renal outcomes in DKD (27, 28). In addition, our study also confirmed that serum ALP, serum UA, and monocyte count were positively correlated with the progression of advanced DKD, whereas platelet count and serum bicarbonate were negatively correlated. Therefore, serum creatinine, age, hemoglobin, serum urea, serum ALP, serum UA, platelet count, serum osmolality, serum bicarbonate, and monocyte count contribute significantly to the LR model. The combined use of these ten variables is expected to provide better efficacy in predicting the occurrence of advanced DKD than using a single biomarker.

Compared with most previous studies, our research offers distinct advantages. Firstly, the variables included in our model are all standard clinical examination items, routinely collected during outpatient or inpatient visits, which are easily obtainable. This provides a feasible foundation for the promotion and application of this model in clinical practice. Furthermore, ML models are often regarded as “black boxes” due to the lack of interpretability in their prediction processes, which may cause clinicians to be cautious about their application and hesitant to make medical decisions based on information that lacks transparency (29). The key contribution of this study lies in the use of SHAP and PDP methods to interpret the prediction logic of ML models both globally and locally, offering a clear explanation of how personalized input data is utilized to make individualized predictions for specific patients. More importantly, by employing the Shinyapps platform, we have integrated this prediction model into a user-friendly online interface, providing significant convenience for clinicians and patients.

Although our research has yielded several notable advances, it is important to acknowledge that certain limitations remain and require further improvement. First, as this study is a retrospective cross-sectional analysis, the research subjects are primarily derived from hospital retrospective EMRs, which introduces the potential for selection bias. Additionally, the study only provides cross-sectional data at specific time points and does not capture the dynamic changes in disease progression over time. Future research should incorporate prospective cohort studies to establish a multi-stage research design that can validate the findings and elucidate causal relationships. Second, the model was developed using data from the Chinese population. Although it underwent external validation using data from the NHANES in the United States, this does not fully substantiate the model’s applicability to other populations. The model variables are based on routine demographic and laboratory tests, and the exclusion of variables such as imaging examinations, lifestyle factors, and social and family backgrounds further limits its performance. Future research should aim to incorporate additional variables and expand the study to multi-regional and multi-center settings to enhance the model’s generalizability. Furthermore, our study contains some missing data. Although multiple imputation methods were applied to address this issue, the absence of complete data may still impair the model’s ability to accurately capture the underlying distribution and characteristics of the data, thus potentially reducing prediction accuracy. Therefore, future studies should focus on increasing the volume of data collection to ensure data integrity and minimize model prediction bias.

In conclusion, this study developed and validated a LR model for predicting advanced DKD, which demonstrated excellent performance in both internal and external validation, indicating strong potential for clinical application. To facilitate clinical use, the LR model has been deployed as a user-friendly web application, accessible to clinicians at the following URL: https://dev2333.shinyapps.io/logistics1/. Nevertheless, this study has certain limitations, particularly regarding sample representativeness and study design constraints, which should be addressed in future research.

## Data Availability

The raw data supporting the conclusions of this article will be made available by the authors, without undue reservation.
